# Risk of future cardiovascular disease in women with prior preeclampsia: a focus group study

**DOI:** 10.1186/1471-2393-13-240

**Published:** 2013-12-21

**Authors:** Ellen W Seely, Janet Rich-Edwards, Janet Lui, Jacinda M Nicklas, Aditi Saxena, Eleni Tsigas, Sue E Levkoff

**Affiliations:** 1 Division of Endocrinology, Diabetes, and Hypertension, Brigham and Women’s Hospital, 221 Longwood Ave, Boston, MA 02115, USA; 2 Harvard Medical School, 25 Shattuck St, Boston, MA 02115, USA; 3 Connors Center for Women’s Health and Gender Biology, Brigham and Women’s Hospital, 1620 Tremont Street, Boston, MA 02120, USA; 4 Division of General Internal Medicine, University of Colorado School of Medicine, 12348 E. Montview Blvd., Aurora, CO 80045, USA; 5 Preeclampsia Foundation, 6767 N. Wickham Road #400, Melbourne, FL 32940, USA; 6 University of South Carolina College of Social Work, 902 Sumter Street Access, Columbia, SC 29208, USA

**Keywords:** Preeclampsia, Cardiovascular disease, Lifestyle change, Web-based intervention

## Abstract

**Background:**

A history of preeclampsia is a risk factor for the future development of hypertension and cardiovascular disease (CVD). The objective of this study was to assess, in women with prior preeclampsia, the level of knowledge regarding the link between preeclampsia and CVD, motivators for and barriers to lifestyle change and interest in a lifestyle modification program to decrease CVD risk following a pregnancy complicated by preeclampsia.

**Methods:**

Twenty women with a history of preeclampsia participated in 5 phone-based focus groups. Focus groups were recorded, transcribed, and analyzed. Qualitative content analysis was used to identify common themes across focus groups. Consensus was reached on a representative set of themes describing the data.

**Results:**

Women with prior preeclampsia were in general unaware of the link between preeclampsia and future CVD but eager to learn about this link and motivated to achieve a healthy lifestyle. Major perceived barriers to lifestyle change were lack of time, cost of healthy foods and family responsibilities. Perceived facilitators included knowledge of the link between preeclampsia and CVD, a desire to stay healthy, and creating a healthy home for their children. Women with prior preeclampsia were interested in the idea of a web-based program focused on lifestyle strategies to decrease CVD risk in women.

**Conclusions:**

Women with prior preeclampsia were eager to learn about the link between preeclampsia and CVD and to take steps to reduce CVD risk. A web-based program to help women with prior preeclampsia adopt a healthy lifestyle may be an appropriate strategy for this population.

## Background

Preeclampsia complicates approximately 5% of all pregnancies and is associated with significant risks to the mother and the fetus [1]. Although preeclampsia resolves following delivery, women with prior preeclampsia have increased long term risk for hypertension (4-fold increased risk) and cardiovascular disease (CVD) (2-fold increased risk) [2,3]. For this reason, the American Heart Association (AHA) now lists history of preeclampsia as a CVD risk factor for women [4]. Furthermore, the AHA recommends that women with a history of preeclampsia undergo lifestyle modification to reduce CVD risk, including smoking cessation, weight reduction, a Dietary Approaches to Stop Hypertension (DASH)-like diet, and increased physical activity.

The objective of this study was to assess knowledge of the link between preeclampsia and CVD, facilitators for and barriers to a lifestyle modification program to decrease CVD risk following pregnancy complicated by preeclampsia, and interest in a web-based lifestyle modification program in women with prior preeclampsia.

## Methods

Women, ages 18–50 years with a pregnancy complicated by preeclampsia within the past 5 years, were recruited to participate in phone-based focus groups. A focus group design was chosen for its potential to be richly hypothesis-generating and a strong tool for exploration of attitudes, beliefs, and intervention development [5,6]. Given the difficulty that many postpartum women face in attending face-to-face focus groups [7], the focus groups were teleconference-based. Phone-based focus groups also enabled inclusion of women in geographically dispersed areas across the United States (U.S.) [8,9]. Focus groups were limited to 4–5 participants to allow for greater in-depth discussion. This size is consistent with research that suggests that when holding teleconference focus groups, smaller groups allow the most meaningful interactions [10]. We held 5 focus groups at which point we reached saturation. This is consistent with focus group research in which the average focus group project consists of 4 to 6 groups, after which the group’s discussion is repetitive, and the researcher reaches data saturation [11].

Women were recruited by postings on the Preeclampsia Foundation website and Facebook page (for 6 days) and Craigslist postings in the major U.S. cities (for 14 days) that directed them to contact the study coordinator by telephone or email. Women who confirmed that they were diagnosed with preeclampsia by a clinician were provided details about the study. Women provided verbal consent to participate, via telephone, to the study coordinator after description of the study protocol. Women were then offered daytime and evening options for a phone-based focus group. The study coordinator sent a factsheet containing the purpose of the study, risks and benefits of participation, a statement of confidentiality, and contact information by email or regular mail per participant preference. Demographic data including age, race and ethnicity, state of current residence, number of pregnancies, number of children, smoking status, and family history of CVD, were collected by a questionnaire administered verbally on the telephone. Participants’ states of residence were categorized by U.S. Census Bureau defined regions [12].

A structured guide was developed to elicit discussion of: knowledge of preeclampsia at the time of the pregnancy complicated by preeclampsia; knowledge of, and reaction to, the association of future CVD risk after a pregnancy complicated by preeclampsia; barriers and facilitators to lifestyle change postpartum; interest in a web-based lifestyle modification program to decrease CVD risk; and preferred timing and mode of delivery of such a program.

A total of five 90 minute focus groups took place in November and December 2012 via teleconference (2 daytime and 3 evening). The focus groups were facilitated by a trained focus group leader with experience conducting prior focus groups [7]. The phone-based focus groups were limited in size per published recommendations [9]. Participants were asked to use their first name only to maintain confidentiality and again provided verbal consent to participate. They were informed that they could leave the call at any time. The focus groups were recorded and transcribed verbatim for analysis (Babbletype, LLC). Participants received a $10.00 gift card and the Preeclampsia Foundation’s ‘Preeclampsia and Heart Disease’ pamphlet [13] after participation. The study was approved by the Brigham and Women’s Hospital Institutional Review Board.

### Data analysis

Data were analyzed following guidelines by Strauss and Corbin (1998) and Miles and Huberman (1994) [14,15]. Transcripts were independently read and re-read by two authors (EWS and SEL) and marked for representative themes that reflected the data [14]. Themes were identified for coding based on: the repetition of specific words, phrases, and opinions; the use of language and general thought patterns, and topics that dominated the focus group discussions. Consensus was reached on representative themes.

## Results

Sixty-four women expressed interest in the study within 8 days of the Craigslist posting and 1 day of the Preeclampsia Foundation posting, of which 30 women were screened for participation. Of these 30 women, 6 were ineligible and 24 were scheduled to participate. Four women who were scheduled to participate did not, one due to poor phone connection. In sum, twenty women participated in 5 focus groups. There were an additional 34 women who expressed interest in study participation but were not enrolled as saturation had been reached. Demographics of study participants are provided in Table 1. Participants called from various locations to participate in the focus group: 16 from home, one from work, one from a family member’s home, one from the parking lot of a library, and one declined to answer.

**Table 1:** Demographics of focus group participants (N = 20)

**Characteristic**	
Age in years, mean (SD)	31.9 (5.6)
**Geographic distribution:**	
Northeast, N (%)	8 (40%)
Midwest, N (%)	4 (20%)
South, N (%)	4 (20%)
West, N (%)	4 (20%)
**Ethnicity:**	
Hispanic, N (%)	0 (0%)
Not hispanic, N (%)	19 (95%)
Declined to answer, N (%)	1 (5%)
**Race:**	
White, N (%)	16 (80%)
Black, N (%)	2 (10%)
Asian, N (%)	1 (5%)
Declined to answer, N (%)	1 (5%)
**Smoking status:**	
Never, N (%)	15 (75%)
Past, N (%)	4 (20%)
Declined to answer, N (%)	1 (5%)
Years since last preeclamptic pregnancy, median (range)	1.5 (.5-5 yrs)
Women with preterm (<34 wks) preeclampsia, N (%)	14 (70%)
Women with >1 preeclamptic pregnancies, N (%)	4 (20%)
Women with twin pregnancies, N (%)	2 (10%)
Women currently pregnant, N (%)	1 (5%)
Number of children, median (range)	1 (0–4)
Age of children, median (range)	2.5 yrs (5 mo.- 20 yrs)

Themes are provided below according to the major domains explored in the focus groups.

### Participant knowledge of preeclampsia at time of pregnancy

The majority of the participants stated that they did not feel that their clinical providers fully explained preeclampsia during their pregnancy: “It was not explained to me what preeclampsia was… I had no idea what was happening until my son was born a week later”. “They told me I had it, but they didn’t really explain… I feel like I had to do most of the research on my own”. “[my provider] said, ‘Preeclampsia is hypertension in pregnancy. There’s no cure except for delivery. That’s why you need to start preparing down that pathway.’ That’s all I remember…”. Several participants indicated that they actively sought information on preeclampsia from outside sources, particularly the internet. Three of the 4 women with recurrent preeclampsia expressed that preeclampsia was explained to them only prior to and/or during the subsequent pregnancy. One of these woman obtained knowledge of preeclampsia prior to her first complicated pregnancy because of elevated blood pressures during infertility treatment.

### Participant knowledge of the link between preeclampsia and future CVD

The majority of participants stated that they had no prior knowledge of the link between preeclampsia and future CVD, and they first learned of this link during the focus group: “No discussion [with my doctor] what [preeclampsia] meant for my own health down the road”. “Nothing was ever explained to me. I had no idea that there might be any long-term side effects… Even five years later, I don’t really know what long-term effects I might expect”. A few women knew about the link from online reading. “I was never told that there could be heart issues, or … cardiovascular concerns down the road. I just recently am finding that information out”. Few women indicated that their clinician had discussed long-term CVD risks associated with preeclampsia. A key focus was concern about impact of preeclampsia on their child’s future risk for high blood pressure or CVD (see below).

### Participant reaction to link of preeclampsia and future CVD

Participant reactions to learning about the link between preeclampsia and future CVD varied, including feeling motivated, empowered, scared, angry, guilty, and isolated: “I think it’s motivating to hear this, because it just encourages me to … be at a healthy weight and do the things that would prevent this kind of thing, instead of just saying, ‘Oh well, I’m going to get it…’” Fear was expressed: “It’s pretty scary especially because if our doctors aren’t talking to us about this… I don’t know what to look for in terms of heart disease.” Some participants saw awareness as a mixed bag. “On one hand, it is good … that I really have to pay attention to the things that I’m doing… But … because I’m only 30 years old, it does stink… because it’s like, ‘Oh well, if it would pop up, when is it going to pop up?’”.

Many participants expressed guilt about their pregnancy complicated by preeclampsia, particularly early delivery and prematurity, and over the potential impact on their children’s future health. “As a mom, you want to protect your children and give them everything you can. By having this disease, I wasn’t able to keep him in long enough for his lungs to fully develop”. “[…it] scares me that my son could be predisposed to having high blood pressure simply because … [he] was born of a mother who had preeclampsia”.

A dominant theme was a feeling of isolation in not knowing other women with a history of preeclampsia, and participants indicated that having a community of other women with a shared pregnancy history would be empowering: “I didn’t know anyone else who had had the condition. I was searching trying to seek out someone who has that. My husband, who’s wonderful, …said to me, ‘Well, I think this all went pretty well.’ I was, ‘Are you absolutely kidding me?’… Having other women to talk with might have been helpful…”. “There’s strength in numbers. Anytime there’s a group available, you can learn from other women’s experiences”. “It would be great to go somewhere and have that support system of other people that have been through your same situation and understand what you’ve been through… it’s like your other family. You share the same experience”.

Women talked about knowledge of the link of preeclampsia and future CVD as a good but underused warning system. “It’s not a very good early warning system if the medical community doesn’t tell us …since most of us didn’t know that it had long-term effects. It’s a good warning system now that we know, but most of the women who have it probably don’t understand that they could have the long-term effects”.

Many participants indicated that the postpartum period after diagnosis of preeclampsia provided an opportunity to incorporate healthy lifestyle changes. Many expressed that their preeclamptic pregnancy was the first time they had been faced with a major health issue: “…because preeclampsia is often such a serious issue…It’s very much an experience that you come out of and say,‘Whoa. What do I do now?’ That’s the best time for an intervention because you’re searching for answers; you’re searching for some reasons as to why this happened”. One participant expressed “… I’ve never maintained what doctors would consider a healthy BMI. I’m now taking it much more seriously”. Another stated “…after my pregnancy, I definitely started more healthy eating and exercising. … knowing that I had preeclampsia had a lot to do with it”.

### Interest in participating in a lifestyle intervention program after a pregnancy complicated by preeclampsia

A major theme was interest in participating in a lifestyle modification program to reduce their own and their children’s risk for CVD. “[I could use] a 12 step program on how to reach your goals… how to reach better health and get to where you want to be physically”, “Tell me what I can change and that gives me some control over my fate, and that feels like more power for me”, and “[if a] program was built for someone like me, with my medical history, I love it”. There were some women who indicated that that they were already leading as healthy a lifestyle as possible regarding diet and exercise. For example, they responded: ”I made a lot of changes after my daughter was born, just on my own, to lose weight and to revamp my diet… we exercise more now, we’re a lot more active than we used to be…“ and “I have completely changed the way I live my life. … I’ve started exercising pretty much every day”.

### Barriers and facilitators to making healthy lifestyle changes after a pregnancy complicated preeclampsia

#### Barriers

Time Constraints: The major barrier to making healthy lifestyle change was time constraints- “There’s so little time to focus on exercising and eating more healthier. Sometimes, I don’t even get a chance to eat until late at night because I’m so busy”, “…I wish that there were 35 hours in my day… just because then I could get everything done” and “I work full time and two kids… I try to figure out when I’m going to take a shower today. Trying to figure out when I’m going to work out is not even on my list at all”.

Cost: “….some women can’t afford to go to the gym. Some women cannot afford to go and buy all healthy fruits and vegetables… there’s definitely some barriers … and budget is a huge part of it”. “Some people are not fortunate enough to buy or go get vegetables”.

Family responsibilities: “You devote so much time to your new family that you get thrown by the wayside in some ways. You’re, ‘Oh, I’ll take care of that later.’ All the time and attention goes especially to your new baby, if not the rest of your family. Then, saying, ‘Oh, when am I going to exercise?’ It’s, ‘Oh, I don’t know’”. “Especially in the first year, it’s so difficult when you’re trying to juggle a baby and meds and doctors’ appointments…”.

Lack of knowledge on healthy diet: “A lot of the times, it’s just not knowing… if you don’t know what foods you should eat, if you want to change you may not be able to” and “There are resources out there on the internet but Googling can take you down rabbit holes of conflicting information and can be difficult to know what sources to trust and what to do”.

Transition of health care: The transition from obstetrical or perinatology provider to primary care provider was identified as a gap in care. “All right, so when I left the hospital, they basically said,’Well, you’re in the hands of your OB.’ Six weeks after when the OB cleared me, she was, ‘All right. Well, now just go talk to your PCP.’ My PCP never said anything about this to me either” and “My perinatologist told me about the long-term risks. I guess now the biggest thing is when I had to find an internal medicine doctor for care… I had to make sure he knew about the risk”. Another participant expressed similar views, saying “we have to advocate for ourselves as patients that have had preeclampsia and try to seek out the research and the doctors that are going to be focused on our care and what we need”.

#### Facilitators

Knowledge: Having the knowledge of the link between preeclampsia and future CVD risk was a key facilitator. “Knowing the long-term impacts of things can make a big difference” and “Anybody who knows they’re at risk would take advantage of programs…to better their health”.

Children: Children were a top motivator for making healthy changes for themselves and their families: “That’s a great side effect, making changes in your lifestyle that it might rub off on your family”, “I’m certainly much more likely to do things to help my kids than I am to help myself”, “I think something else that might be helpful …is knowing [how] healthy lifestyle can affect your whole family” and “One of my biggest motivations, and I’m sure it’s for everyone, is my child, both having her see a healthy lifestyle and have me be around for her”.

### Preferred design and timing of a lifestyle modification program

An online lifestyle modification program was preferred because of its flexibility, given the difficulty of attending programs outside home or work. Women expressed a high level of access to the internet and interest: “I like the online option…I’m always on the internet…it’s something I could access whenever I have free time and get more information”. “Just because it’s something that I could access at any point in my day, whether it would be evening or night or morning.” and “I am busy, but I do always have access to something online, either my computer or my iPad or my iPhone”. Some also wanted the opportunity to meet with women with a similar pregnancy history in addition to the online program. “I think that having some sort of an online community is good and certainly very helpful. But would there be any sort of face-to-face component…? Because I think that’s a really important thing too.”

Variability existed as to the best time to initiate a postpartum lifestyle modification program. “Those first six weeks are obviously so busy. I think just around that six weeks that it would be a good time to introduce an intervention”, “You need a couple of months to just settle in and try to figure things, your new schedule out” and “…for every woman it’s going to be different… depending on your medical condition and when your doctor allows you to start exercising”. “…it’s case by case basis because I think some women might be ready for it right out of the hold. I think some women might not be but they want it later”.

## Discussion

This study demonstrates that women with prior preeclampsia are often unaware of their increased risk for future CVD and are eager to learn about the link. Perceived individual level barriers for postpartum lifestyle change included lack of time, family responsibilities, lack of knowledge about healthy diet, and higher cost of healthy foods. System level barriers included the gap in transition from obstetric/perinatal to primary care, with participants emphasizing the importance of finding a primary care physician who was knowledgeable about their increased risk for CVD. Perceived facilitators to healthy lifestyle adoption included knowledge about the link between preeclampsia and future CVD, use of the “window of opportunity” following a pregnancy complicated by preeclampsia, education about healthy eating, and access to a community of women with recent preeclampsia. Women identified impact on the family, especially their children, as a major motivator for lifestyle change. Participants expressed a high level of enthusiasm about a postpartum lifestyle modification program. They perceived multiple advantages to an online program developed expressly for women like themselves, which would allow them access whenever they had free time available, and provide opportunities to connect with others who had experienced a similar pregnancy.

Results of these focus groups share common features as well as differences with the focus group and informant interview study we performed in women with a history of gestational diabetes mellitus (GDM) where women were recruited via fliers and through doctors’ offices [7]. Similar motivators included the potential for healthy changes to impact the entire family and the postpartum period as a “window of opportunity” for lifestyle change. Similar barriers included lack of time. Women in both populations discussed their guilt about having the condition and about how it might affect their children. While most women with prior GDM were aware that a pregnancy complicated by GDM increased their future risk for type 2 diabetes, most women with prior preeclampsia had no knowledge of the link with future CVD, with some hearing of the link for the first time during the focus groups. Both populations were interested in a lifestyle modification program delivered via the web. However the desire for contact with a community of similar women was a prominent theme for women with a history of preeclampsia whereas it was not in our study of women with recent GDM. Given that women with GDM are typically diagnosed at 24–28 weeks of pregnancy, they have 12–14 weeks of outpatient care during which they can meet other women with GDM in their care setting or in their communities. Women with preeclampsia often have an abrupt diagnosis resulting in hospitalization and bed rest and/or delivery where they are often isolated from other women with this diagnosis.

Similar to our findings, other studies found poor knowledge and confusion about the implications of preeclampsia [16,17]. One study with face-to-face interviews found increased knowledge of preeclampsia was associated with a prior preeclamptic pregnancy [16]. Similarly in our study, the few women who indicated that they had been educated about preeclampsia were those who had experienced preeclampsia in a prior pregnancy. Also consistent with our findings, a UK-based study showed that women expressed confusion about the future implications of preeclampsia, and concern over the future health of their child, rather than their own health [17]. Other studies found similar interest in lifestyle counseling programs, and identified similar barriers and motivators to lifestyle change [18]. In a Netherlands-based focus group study of 36 women with pregnancies complicated by preeclampsia, intrauterine growth restriction, and/or GDM, [18,19] motivators to healthy lifestyle included: experiencing a complicated pregnancy; desire to prevent recurrence of the condition in a subsequent pregnancy, and to be a good role model for their children [19].

We used teleconference focus groups rather than face-to-face focus groups based on our prior finding that postpartum women found it difficult to attend scheduled face-to-face focus groups [7]. Teleconference focus groups allowed greater flexibility in being able to participate from home or work, obviating travel time and potentially the need for and expense of childcare. In addition, we were interested in having women from different geographic areas of the U.S. participate in the focus groups, which would have not been possible with a face-to-face group. Although teleconference focus groups have a potential disadvantage of omission of body language cues, there may be greater honesty in opinions expressed because of the diminution of peer pressure experienced in a face-to-face group [20].

There are several potential limitations to this study. The majority of women who participated in the focus groups were Caucasian, and the findings may not apply to other ethnic/racial groups. A history of preeclampsia was obtained by self-report recall of clinician diagnosis. A review of studies of maternal recall of hypertension in pregnancy reported a specificity ranging from 96% to 100% for maternal recall of preeclampsia [21]. Another study demonstrated a 98% specificity for maternal recall of preeclampsia against medical record validation using International Society for the Study of Hypertension in Pregnancy criteria [22]. Of interest, a graphic education tool was shown to improve patient knowledge of preeclampsia during pregnancy [23]. Whether such a tool would improve the accuracy of maternal recall of diagnosis after pregnancy is unknown at this time. During the focus groups for our study, women provided details about their pregnancies, supporting the accuracy of the diagnosis of preeclampsia. A large proportion of women in our focus groups reported preterm preeclampsia and results of focus groups of women with term preeclampsia may differ. In addition, given that women in our study were recruited via web-based methods, they may have been more positive about web-based lifestyle modification programs than other populations. However, we found similar interest in a web-based lifestyle modification study in a GDM population recruited via doctor’s offices and fliers [24].

## Conclusions

There are several implications of our study findings for care of women with prior preeclampsia both at the individual and system levels. The majority of women with prior preeclampsia were unaware of the link between preeclampsia and future risk of CVD. Once aware of this link, participants saw the period after their pregnancy as a potential “window of opportunity” for adopting healthy lifestyle behaviors to reduce future CVD risk. Clinicians should take steps to minimize gaps in care between obstetrics and primary care, and ensure that they educate women with prior preeclampsia about their increased risk for CVD.

Given the findings of this study, programs for CVD risk reduction that take advantage of the postpartum “window of opportunity” for women with prior preeclampsia need to be developed. Web-based programs offer the potential of reaching this population and providing a mechanism to overcome time barriers and knowledge gaps, as well as to decrease isolation by providing a connection to other women with a history of preeclampsia.

## Abbreviations

AHA: American Heart Association; CVD: Cardiovascular disease; DASH: Dietary Approaches to Stop Hypertension; GDM: Gestational diabetes; U.S.: United States.

## Competing interests

The authors report no conflicts of interest. The authors alone are responsible for the content and writing of the paper.

## Authors’ contributions

EWS, JRE and SEL conceived of and designed the study. EWS and SEL read the focus group transcripts and derived themes and co-wrote the manuscript. JL and ET participated in recruitment of the subjects. EWS, JRE, JL, JMN, AS, ET and SEL reviewed, edited and contributed to the manuscript. All authors read and approved the final manuscript.

## Pre-publication history

The pre-publication history for this paper can be accessed here:

http://www.biomedcentral.com/1471-2393/13/240/prepub
